# Implications of Environmental Variations on *Saccharina japonica* Cultivation in Xiangshan Bay, China

**DOI:** 10.3390/biology14020175

**Published:** 2025-02-09

**Authors:** Yikang Bao, Peng Xu

**Affiliations:** College of Oceanography and Ecological Science, Shanghai Ocean University, Shanghai 201306, China; ykb8196@163.com

**Keywords:** suspended kelp cultivation, Xiangshan Bay, mariculture, environments, macroalgae

## Abstract

This study took Xiangshan Bay as an example to illustrate the variation characteristics of the physicochemical environments (temperature, salinity, light, nutrients, and currents) during one kelp cultivation cycle. The study was conducted from November 2020 to May 2021 through tracking down observations. Furthermore, the environmental factors were evaluated using suitability functions of kelp growth, aiming to provide references for promoting kelp cultivation in South China. We discussed the self-limiting effect of nutrients in the culture zone. The results showed that the average temperature, salinity, and light intensity during the cruises in Xiangshan Bay kelp farm were characterized by seasonal variations. Temperature was found to be the most critical environmental factor in determining the kelp cultivation window and hence the yield in Xiangshan Bay. The dissolved inorganic nitrogen (DIN) concentrations initially decreased and then increased, while the dissolved inorganic phosphorus (DIP) concentrations remained decreasing along with the kelp cultivation. The surface tide currents were dramatically attenuated by the suspended kelp cultivation, while the quasi-steady circulations which played a key role in nutrient supplementation for kelp cultivation were not weakened by the kelp cultivation. Among the cruises, the suitability indices’ ranges for temperature, salinity, light, and nutrients were 0.02–0.94, 0.96–0.99, 0.97–1, 0.96–0.97 (DIN), and 0.92–0.95 (DIP), respectively. The results of the suitability functions demonstrated that the salinity and light conditions in Xiangshan Bay were very suitable for kelp cultivation and would not cause significant cultivation risks. Although the cultivated kelp could greatly absorb nutrients, the suitability index of nutrients remained adequate even during the late stage of cultivation, indicating the present kelp cultivation scale has not reached the carrying capacity of Xiangshan Bay and there is still much potential for development. To this end, further selective breeding of the thermal tolerance variety has become the key to improving the kelp cultivation performance in Xiangshan Bay. Meanwhile, the self-limiting effects in relation to nutrients are not significant in the Xiangshan Bay kelp farm, but it might be more significant in other kelp farms.

## Simple Summary

Climate change has a significant negative impact on human economic activities, including seaweed mariculture. Kelp cultivation plays an important role in global mariculture due to its significant economic value and important ecological services. Currently, kelp is widely cultivated in China’s coastal seas, and the annual yield has increased by approximately 4–5% over the past decade. However, the desired outcomes may not be achieved by blindly increasing the kelp cultivation scale and the cultivation density in one kelp farm, without scientific evaluations of the relationship between kelp cultivation and its environmental variations. Therefore, this study took Xiangshan Bay as an example to elucidate the variation characteristics of the physicochemical environment and its effects on the growth suitability of *Saccharina japonica*. We further explored the key limiting factors restricting the enhancement of kelp cultivation efficiency in Xiangshan Bay. We systematically studied the self-limiting effect in kelp cultivation. These efforts are intended to provide scientific references for improving the cultivation performance.

## 1. Introduction

Changes in environmental characteristics, primarily temperature, affect many physiological processes in different organisms [1,2]. Over time, humans have learned to use the implications of environmental changes in their activities. Macroalgae cultivation holds a prominent position in global mariculture due to its significant economic values and important ecological services [3]. The global yield of cultivated macroalgae reached over 35.1 million tons of wet weight in 2022 [4]. China is the largest producer of cultivated macroalgae, among which the annual yield of kelp was about 1.73 million tons with economic values of about ten billion RMB [5]. Kelp cultivation not only benefits the marine economy but also helps to improve the marine environments such as by mitigating eutrophication and acidification in coastal seas [6,7]. Meanwhile, kelp cultivation contributes to the blue carbon sinks and hence would help China to achieve carbon neutrality [8]. As a consequence, kelp has been widely cultivated in North and South China coastal seas, and the annual yield has increased by approximately 4–5% over the past decade [9].

The rapid development of kelp cultivation calls for scientific guidance, which should be based on a thorough understanding of the relationship between kelp cultivation and the environment. Kelp species cultivated in China is mainly *Saccharina japonica* (Aresch.) C.E. Lane, C. Mayes, Druehl & G.W. Saunders [10] and cold-water [11], and the cultivation period is roughly from the winter to the beginning of the following summer [12]. Kelp seedlings of 2–3 cm need to be temporarily cultured to approximately 20 cm in a horizontal hanging or vertical hanging manner, and then, the kelps are taken for cultivation in the suspended kelp farm [13]. Some researchers argued that the physicochemical factors in the farm could exert significant influences on the kelp (*Saccharina japonica*) growth and hence the yield based on rough observation [11,14,15]. Particularly, they found that a good harvest could be achieved where water exchange was less suppressed by the kelp cultivation [15]. Some researchers obtained quantitative relationships between the kelp growth and the physicochemical factors through laboratory experiments [16,17,18,19,20,21]. Temperature, salinity, light, nutrients, and carbon dioxide were the main physicochemical factors studied by the researchers in the laboratory [20,22,23,24,25,26,27]. The suitable water temperature of *Saccharina japonica* ranged from 0.5 °C to 20 °C, and higher or lower temperatures prevented its growth [11,14]. When the salinity was lower than 3 psu, kelp exhibited negative phosphorus uptake [28]. The most suitable salinity for kelp growth was about 29–32 psu [22]. The highest tolerable salinity for kelp was mostly considered to be 40 psu [28]. Excessive and insufficient light intensity can both inhibit the photosynthesis and growth of *Saccharina japonica* [14]. In realistic kelp farms, carbon dioxide would not usually be regarded as a limiting environmental factor to kelp growth since kelps are cultivated near the air–sea interface [29,30]. On the contrary, ocean currents which are not usually considered in the laboratory play an important role in realistic kelp farms due to their transport effects of nutrients [31]. Strong ocean currents directly cause the shedding of kelp withered edges and more brittle parts [32], while some studies have suggested that it is hard to determine the direct impact of currents on the growth rate of macroalgae such as kelp [33]. Nowadays, it is believed that the effects of currents on kelp growth are reflected in indirect mechanisms, such as mixing and material transport in the water column. These hydrodynamic processes can lead to alterations in nutrient concentrations, which may either promote or inhibit algal growth [34]. Therefore, temperature, salinity, light, nutrients, and currents were the main physicochemical factors explored in in-situ studies in kelp farms.

Some scholars summarized some physicochemical factors’ functions applicable to kelp growth, which could accurately reflect the quantitative relationship between different ranges of physicochemical factors and kelp growth rate [16,17,18,19]. These functions (growth rate curves) could represent the degree of suitability of different ranges of physicochemical factors for kelp growth. Hence, these functions are referred to as suitability functions in the paper. The suitability functions of kelp were integrated into a kelp growth model, which played a fundamental role in modeling studies about kelp cultivation [25,32,35]. However, it should be pointed out that these suitability functions only took the regulation effects of the physicochemical environments on kelp growth into account while ignoring the regulation effects of kelp cultivation on the environments. The regulation effects of kelp cultivation on the physicochemical environments are mainly manifested in two aspects: (1) The cultivated kelp would strongly absorb nutrients and hence decrease the nutrient concentrations [36]; (2) The kelp cultivation would dramatically weaken the hydrodynamic processes dominated by tides in the farm [37]. Some researchers explored assessing the carrying capacity of Sanggou Bay by constructing physical–biological coupling models [38,39], and there were significant self-limiting effects of kelp cultivation in the coupling models, meaning the physicochemical environments regulated by kelp cultivation would limit the performance of kelp cultivation in turn. The research of self-limiting effects during kelp cultivation should not be neglected, especially when scaling up to larger-scale kelp cultivation. In summary, the desired outcomes may not be achieved by blindly increasing the kelp cultivation scale or density in one kelp farm, without scientific evaluations of the effect of physicochemical environment variations on kelp cultivation.

The mature kelp size of the Xiangshan Bay kelp farm is much smaller than that in typical kelp farms of the north [40,41]. It may be attributed to the limitation of some key physicochemical factors. Therefore, this study takes Xiangshan Bay as an example to elucidate the variation characteristics of the physicochemical environment and its effects on the growth suitability of *Saccharina japonica*. Moreover, we explored the key limiting factors restricting the enhancement of kelp cultivation efficiency in Xiangshan Bay and further systematically studied the self-limiting effect in kelp cultivation, to provide scientific references for improving the cultivation performance.

## 2. Materials and Methods

### 2.1. Studies Area

**Figure 1:**
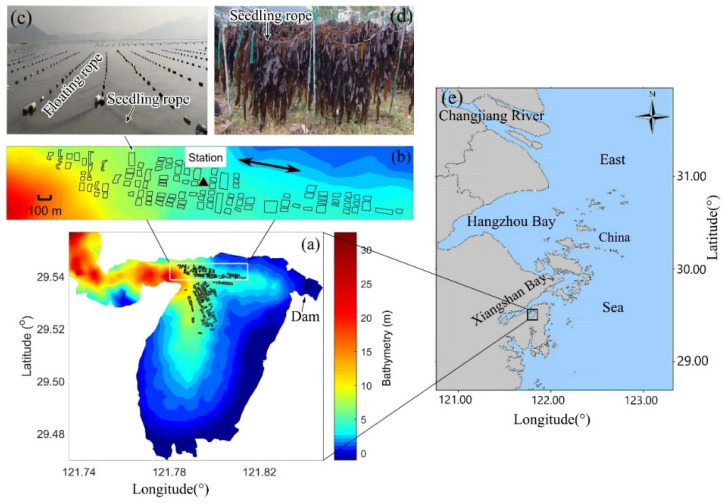
Observation station in Xiangshan Bay. The small black rectangles in (**a**,**b**) represent the suspended kelp farm in Xiangshan Bay. (**b**) is a zoomed-in representation of the white box in (**a**). The observation station is marked with a black triangle in (**b**). The detailed structures of the square suspended rafts are presented in (**c**) consisting of floating and seedling ropes. The seedling ropes in (**c**) are already mounted with kelp seedlings. The harvested kelps are presented in (**d**). Colors in (**a**,**b**) represent bathymetry. The bidirectional arrow in (**b**) represents the flooding and ebbing currents’ directions, respectively. The location of Xiangshan Bay in East China Sea is marked in (**e**).

Xiangshan Bay (29°23′–29°49′ N, 121°25′–122°00′ E) is located in the southeast of Ningbo City, Zhejiang Province, with a kelp farm of about 3 km^2^ (Figure 1a). The floating kelp rafts in Xiangshan Bay consist of floating ropes and seedling ropes (Figure 1c). The floating ropes in Xiangshan Bay are aligned with currents and provide buoyance so that the rafts can be suspended on the sea surface. The seedling ropes perpendicular to the currents are used to hang kelp seedlings (Figure 1c,d). The cultivated kelp species is *Saccharina japonica*, which grows on the surface of Xiangshan Bay. The base of the kelp blades (growing point) is at the water depth of about 0.5 m, and the blades grow downward. The kelp farm in Xiangshan Bay is 4–10 m deep. The hydrodynamics environments in Xiangshan Bay are characterized by strong semi-diurnal tides [42]. Winds and waves are weak in Xiangshan Bay since it is surrounded by hills [43]. The eutrophication in Xiangshan Bay is significant since lots of terrigenous agriculture and industry wastewater flows into the bay during the rainy season [44,45]. The coastlands in South China are dominated by hilly terrain, so there are many small dams to conserve fresh water. A small dam which was built at Xiangshan Bay head (Figure 1a) would release terrigenous fresh water into the bay from time to time when the water level is high, particularly during rainy seasons. The rainy seasons of Xiangshan Bay are from March or April to September [46], and the rest of the time excluding the rainy seasons during one year are dry seasons.

### 2.2. Data Collection and Processing

The tracking down observations were conducted at a station located in the center of the northern part of Xiangshan Bay kelp farm, where the currents are rectilinear (Figure 1). The tracking down observations consisted of five cruises (Table 1), which covered one complete cultivation cycle. The first and last cruises (202011 and 202105 in Table 1) were conducted before the kelps were planted and after the kelps were harvested, respectively. The remaining three cruises (202101, 202102, and 202104 in Table 1) were conducted during the early, middle and late stages of the kelp cultivation period, respectively. Cruises 202011, 202101 and 202102 were conducted in the dry seasons at Xiangshan Bay, while cruises 202104 and 202105 were conducted in the rainy seasons. During each cruise, we obtained four to six kelps from the same location in the Xiangshan Bay kelp cultivation area, and laid them flat on one laboratory table to measure the size of each kelp blade by using a straightedge (with an accuracy of 1 mm). The average plant size of kelp was 137.4 × 37.1 cm at the late stage (Table 1). Each of the five cruises lasted over seven days which covered spring and neap tides. The observation contents of each cruise were similar and included two main parts. One was continuously sampling throughout each cruise by automatic instruments including an Alec electromagnetic current meter (ECM), which collected the surface currents and temperature every half an hour, and a RDI 1200 kHz Workhorse acoustic Doppler current profiler (ADCP) which could provide bottom currents and tide elevation data every half an hour; the other was spring-tide-day (over 24 h) and neap-tide-day (over 24 h) observations during each cruise including manually launching a SBE 19plus conductivity–temperature–depth sensor (CTD) which collected salinity, temperature and light intensity profiles, manually sampling surface water. Surface water samples were manually collected at the tracking down observations station. During each cruise, approximately 50 surface water samples were manually collected at the depth of about 0.5 m. The water samples (100 mL) were added to about 1 mL of Trichloromethane in the field and placed in an ice box, and then, they were analyzed by a Dechem-Tech CleverChem 200 auto discrete analyzer in the laboratory to obtain the DIN (NO_3_^−^, NO_2_^−^, NH_4_^+^) and DIP concentrations. The molybdenum blue method was used to measure DIP, while NO_3_^−^ and NO_2_^−^ were measured using the cadmium reduction method and diazotization, respectively. NH_4_^+^ was determined using the indophenol blue method. All measurements were conducted in triplicate, and blanks were analyzed to account for any contamination. The detection limits for DIN and DIP were 0.05 µmol/L and 0.01 µmol/L, respectively. Sample collection and on-site handling, as well as analytical methods, were conducted in accordance with the ‘Oceanographic Survey Standards’ (GB 17378–2007) [47]. The analysis of data was performed using the software of MATLAB R2018b, EXCEL 2016, and SPSS 24. The means ± standard deviation were derived from the data, and correlations between growth parameters and physicochemical factors were determined with Spearman’s correlation test. Unless stated otherwise, significance refers to *p* ≤ 0.05.

The average values of the temperature, salinity, nutrient concentrations, and currents were employed to represent the status of the cruise. The monthly average light intensity was calculated based on the average light intensity during spring-tide-day and neap-tide-day each of cruise, along with the number of sunny, cloudy and rainy days during the month The Eulerian averaging method was employed to filter the periodic tide currents to obtain the quasi-steady residual circulation which was responsible for nutrient transport [48]. The averaging time window was 12 tide periods during each cruise. It should be noted that the ECM was not deployed during 202105, and the surface temperature and currents data were provided by CTD and ADCP, respectively.

### 2.3. Suitability Assessment Method

The average values of the physicochemical environmental factors collected from each cruise could be substituted into the suitability functions to obtain the suitability indices, which could assess the suitability of the environment for kelp growth [49]. The suitability indices could quantitatively assess whether these physicochemical conditions were favorable or limiting for kelp growth during various cultivation stages. This assessment method enables us to determine the key physicochemical factors that limit kelp cultivation efficiency, as well as the impact of their drastic variations on the suitability of kelp growth. The more the results approached 1, the more suitable the physicochemical conditions were for the kelp growth, and vice versa. The suitability function of temperature is as follows [16]:

(1) $$\begin{array}{c} f(T)=exp\;[-2.3\times ({\frac{T-T_{opt}}{T_{x}-T_{opt}})}^{2}] \end{array}$$

where T represents sea surface temperature, T_opt_ and T_x_ are the optimum temperature for kelp growth and the minimum or maximum temperature threshold for kelp growth, respectively. T_opt_ is 10 °C [35], and T_x_ is 0.5 °C or 20 °C [11,35]. When T ≤ T_opt_, Tx = 0.5 °C. When T > T_opt_, T_x_ = 20 °C. According to the expression for salinity limitation proposed by Martins et al. in the modeling of seaweed, a macroalgae [17], the salinity suitability function for *Saccharina japonica* is adopted when the salinity of seawater is greater than five:

(2) $$f\left(S\right)=1-(\frac{S-S_{opt}}{S_{x}-S_{opt}}{)}^{m}$$

where S represents sea surface salinity, S_opt_ and S_x_ are the optimum salinity for *Saccharina japonica* growth and the minimum and maximum salinity threshold for *Saccharina japonica* growth, respectively. S_opt_ is 30 [22]. S_x_ is 3 or 40 [28]. When S < S_opt_, S_x_ = 3, m = 2.5. When S ≥ S_opt_, S_x_ = 40, m = 2. The light suitability function is mostly adopted as Steele’s formula (optimal curve of photoinhibition model) [18]:

(3) $$f\left(I\right)=\frac{I}{I_{opt}}\times e^{\left(1-\frac{I}{I_{opt}}\right)}$$

where I and I_opt_ represent the surface light intensity and the optimum light intensity for kelp photosynthesis, respectively. The natural constant e was about 2.72. The optimum light intensity is 180 μmol·m^−2^·s^−1^ [24,35]. Some studies suggested that the absorption characteristics of nitrogen and phosphorus by kelp were in line with the saturation absorption dynamics [19]. Therefore, the Michaelis–Menten equation could be used to analyze the effects of variations in DIN and DIP of the sea surface on the growth suitability of *Saccharina japonica*:

(4) $$f\left(DIN\right)=\frac{DIN}{K_{c1}+DIN}$$

(5) $$f\left(DIP\right)=\frac{DIP}{K_{c2}+DIP}$$

where DIN and DIP represent the nutrient concentrations. K_c1_ and K_c2_ stand for the half-saturation constant for DIN and DIP, respectively, which were 1.29 μmol/L and 0.13 μmol/L [19].

## 3. Results

### 3.1. Characteristics of Prominent Physicochemical Factor Variations

#### 3.1.1. Temperature

**Figure 2:**
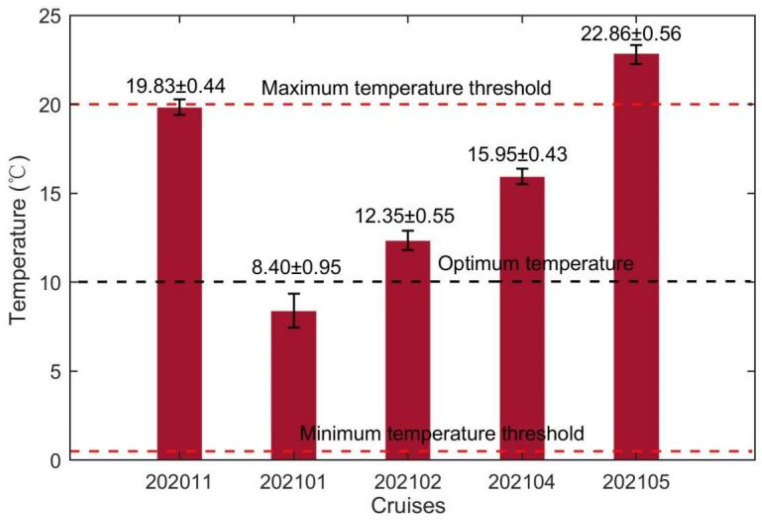
The average sea surface temperature at the observation station during each cruise. The numbers marked above bars are average temperature ± standard deviation. The red dashed lines mark the maximum and minimum temperature threshold for kelp growth, respectively. The black dashed lines mark the optimum temperature for kelp growth.

The average sea surface temperature initially dropped, then increased with every next cruise (Figure 2). The lowest average surface temperature was 8.40 °C, which was collected during 202101 (deep winter). The average surface temperature during all cruises exceeded the minimum temperature threshold for kelp growth. The average surface temperature of cruises 202011 (late autumn) and 202105 (late spring) were 19.83 °C and 22.86 °C, respectively. The average surface temperature during 202011 and 202105 were, respectively, close and higher than the maximum temperature threshold, determining the time window for kelp cultivation in Xiangshan Bay.

**Figure 3:**
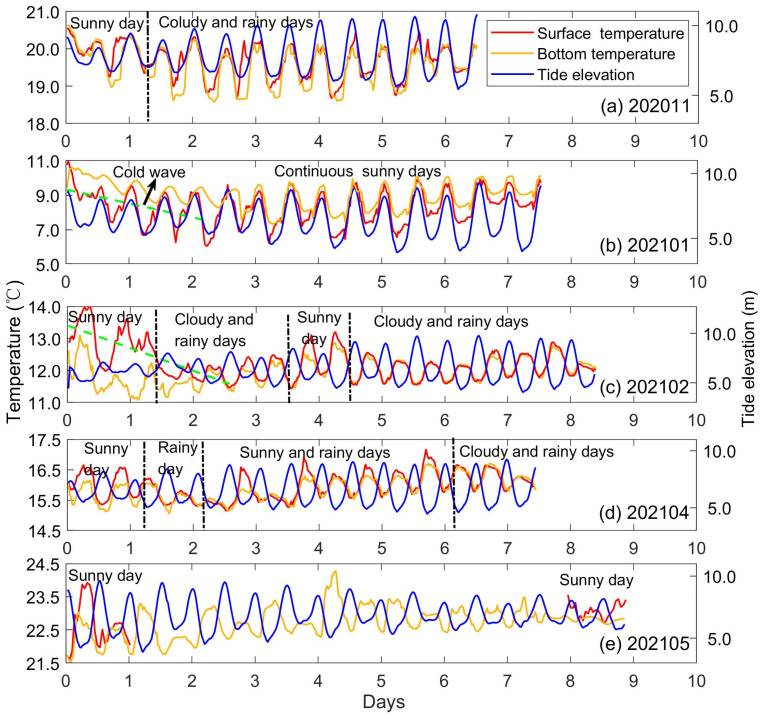
The time series of sea surface temperature (red lines), bottom temperature (yellow lines), and tide elevation (blue lines) during each cruise. The dashed green lines in (**b**,**c**) represent the variation trends of sea surface temperature during that time. The weather conditions are illustrated by words above the time series.

The standard deviation, representing temperature fluctuations during each cruise, was smaller than the average temperature by one to two orders. Therefore, the most prominent variation in the surface temperature in Xiangshan Bay kelp farm was seasonal variation. The standard deviations of sea surface temperature were mainly contributed by temperature fluctuations at weather events, tide, and daily timescales (Figure 3). A cold wave event during 202101 caused a considerable drop of sea surface temperature by 3 °C in 2 days (Figure 3b). Additionally, the transition from continuous sunny days to continuous cloudy and rainy days during early 202102 caused the sea surface temperature to decrease by about 1.8 °C (Figure 3c). Surface temperature not only fluctuated at tide timescale as bottom temperature, but was also regulated by the processes on the air–sea interface, such as weather events as mentioned above. During continuous cloudy and rainy days as in late 202102 (Figure 3c), no significant deviation was identified between the surface and bottom temperature. In contrast, during continuous sunny days as in late 202101 (Figure 3b), deviations between the surface and bottom temperature varied as day and night, indicating there were fluctuations at the daily timescale in surface temperature.

#### 3.1.2. Salinity

**Figure 4:**
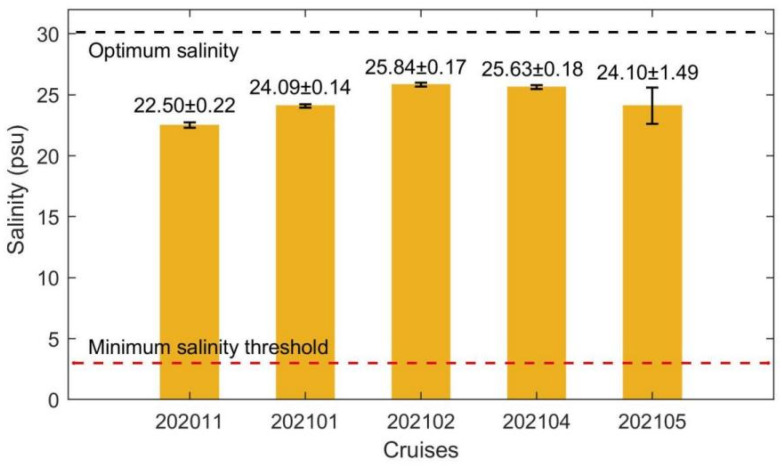
The average sea surface salinity at the observation station during each cruise. The numbers marked above bars are average salinity ± standard deviation. The black and red dashed lines mark the optimum salinity and minimum salinity threshold for kelp growth, respectively.

The average sea surface salinity initially increased and then decreased along with the cruises (Figure 4). The highest average surface salinity, which was 25.84, was recorded during 202102, while the two low peaks of the average surface salinity observed during 202011 and 202105 were 22.50 and 24.10, respectively. Across all cruises, the average surface salinity were slightly lower than the optimum salinity for *Saccharina japonica* growth but much higher than the minimum salinity threshold. The standard deviation, representing the salinity fluctuation, was about two orders smaller than the average salinity during each cruise. Therefore, the most prominent variation in the average surface salinity in Xiangshan Bay kelp farm was seasonal variation which was related to rainy seasons and dry seasons. The salinity continuously increased during dry-season cruises (202011, 202101, 202102) and continuously decreased during rainy-season cruises (202104 and 202105).

**Figure 5:**
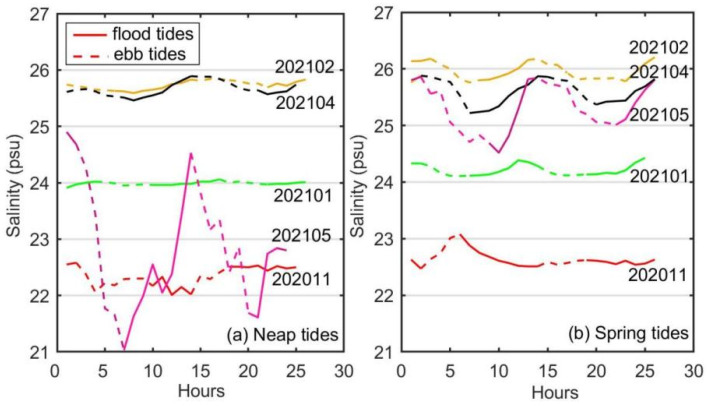
The time series of sea surface salinity during neap-tide-day and spring-tide-day. Different colored lines were used to distinguish various cruises. The solid and dashed lines represent the flood and ebb tides, respectively.

The standard deviations of the surface salinity, which were generally larger during rainy seasons than during dry seasons (Figure 4). The standard deviations or the salinity fluctuations of one cruise revealed the along-bay salinity gradient. Therefore, the along-bay salinity gradients in Xiangshan Bay kelp farm were stronger during rainy seasons than during dry seasons, and could reverse directions under some conditions such as during 202011. The fluctuations of the surface salinity during the 202105 neap tides were particularly larger than those during the 202105 spring tides. Considering there was no heavy rain during 202105, the dramatic drop of the surface salinity during 202105 neap tides was attributed to the release of terrestrial water from the dam at the bay head (Figure 1), giving rise to larger fluctuations (Figure 4 and Figure 5).

#### 3.1.3. Light

**Figure 6:**
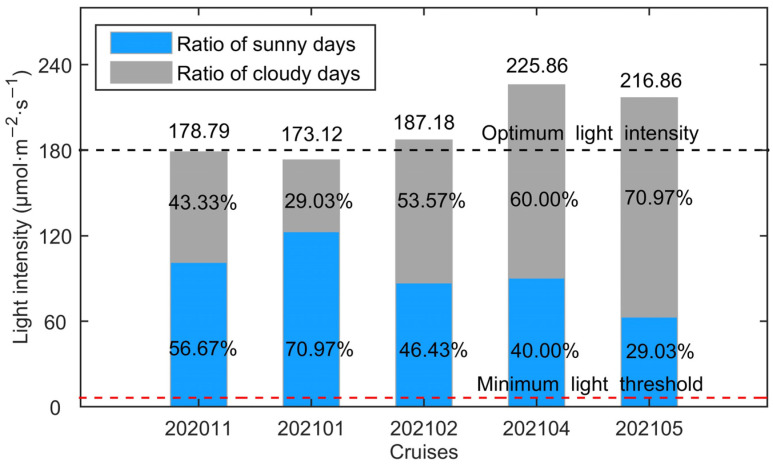
The average light intensity at the observation during each cruise. The numbers marked above bars are the average light intensity. The gray and blue parts in a bar represent the time ratios of sunny days, and cloudy and rainy days in the cruise month, which are marked by the percent numbers. The black and red dashed lines represent the optimum light intensity and minimum light threshold (6.21 μmol·m^−2^·s^−1^) [14] for kelp growth, respectively.

The average surface light intensity during 202011, 202101, and 202102 which were conducted during dry season (when the ratio of cloudy days in the month ranged between 29 and 54% according to our observations) presented a trend of initially decreasing and then increasing along with the cruises (Figure 6). The weakest surface light intensity was found during 202101 with a value of 173.12 μmol·m^−2^·s^−1^. The surface light intensity during 202011 and 202102 was stronger, with values of 178.79 and 187.18 μmol·m^−2^·s^−1^, respectively. When the rainy season came, the ratio of cloudy days increased. The surface light intensity during 202104 was strongest among the cruises, reaching about 225.86 μmol·m^−2^·s^−1^. The surface light intensity during 202105 was a little lower than 202104 since the ratio of cloudy days became higher.

#### 3.1.4. Nutrients

**Figure 7:**
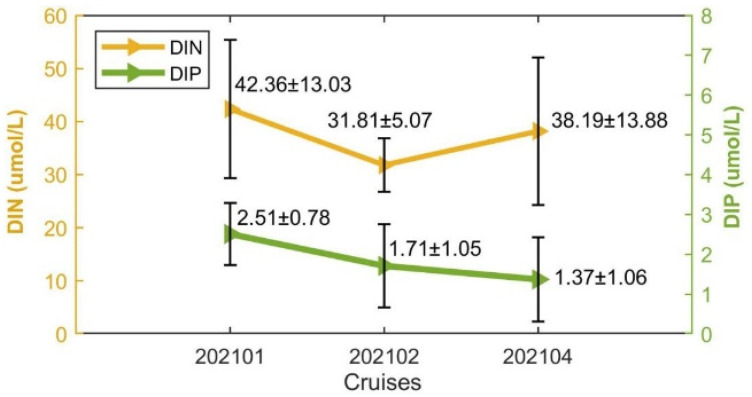
The concentrations of DIN and DIP during cruises when kelps were cultivated. The numbers marked close to the triangles are average nutrient concentrations ± standard deviation.

The average DIN concentrations initially decreased and then increased during the kelp farming period (Figure 7). The highest average DIN concentration was 42.36 μmol/L collected during 202101, which then decreased to 31.81 μmol/L during 202102 and finally increased to 38.19 μmol/L during 202104, which was still lower than that during 202101. Unlike DIN, the average concentrations of DIP remained decreasing throughout the kelp farming period, with values of 2.51 μmol/L, 1.71 μmol/L, and 1.37 μmol during 202101, 202102, and 202104, respectively. The different variation trends between DIN and DIP during the second half-cultivation period might be attributed to the terrigenous agriculture and industry wastewater loaded into the bay as characterized by a high N/P ratio [50]. The standard deviations of the DIN and DIP concentrations during each cruise accounted for approximately 27.70% and 56.60% of the average concentrations on average (Figure 7), respectively. The standard deviations of DIN and DIP concentrations could reveal the along-bay gradient of nutrient concentrations under the advection of tide currents as salinity. Therefore, the considerable standard deviations of DIN and DIP concentrations revealed the great absorption effects of nutrients by the cultivated kelp.

#### 3.1.5. Currents

**Figure 8:**
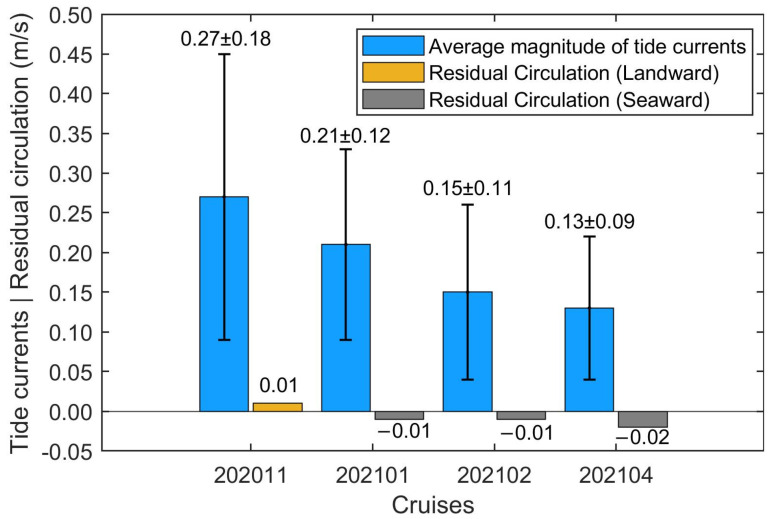
The average magnitudes of the surface tide currents (blue bars) and the surface circulations (yellow and gray bars). The numbers marked above blue bars are the average ± standard deviation of magnitudes of the surface tidal currents. The numbers marked above yellow and gray bars represent the circulation during each cruise. Note the positive circulation is landward while the negative ones are seaward.

The average magnitude of surface tide currents for each cruise in Xiangshan Bay kelp farm is shown in Figure 8. The average magnitude of the surface tide currents was 0.27 m/s during 202011 without the suspended kelp cultivation. When the kelp seedlings were planted in 202101, the average magnitude of surface tide currents decreased by about 22.23% to 0.21 m/s. When the kelps grew to about mature size in 202102 and 202104, the average magnitudes of surface tide currents further decreased by about 44.44% and 51.86% compared to 202011, to the values of 0.15 m/s and 0.13 m/s, respectively. Meanwhile, the standard deviation of surface tidal currents decreased along with cruises (Figure 8), indicating the damping effect of tide waves caused by the suspended kelp cultivation.

The surface circulations after filtering periodic tide currents based on the Eulerian averaging method are presented in Figure 8. The surface circulations in 202011, 202101, 202102 and 202104 were about 0.01 m/s, −0.01 m/s, −0.01 m/s and −0.02 m/s, respectively. The surface circulation during 202105 was not calculated since ADCP fell over 1.5 days after the deployment. It should be noted that the surface circulation reversed during the tracking down observations. The surface circulation was landward (positive) in 202011, while it was seaward (negative) in 202101, 202102 and 202104. The turning of the circulation directions from 202011 to 202101 might be caused by the reverse of the along-bay salinity gradient which drove the gravity circulation. Among the cruises, the surface circulation was strongest during 202104 in the rainy season and the *Saccharina japonica* grew to its mature size.

As shown in Table 2, the length of kelp exhibited significant positive correlations with surface temperature and salinity, while it showed significant negative correlations with surface DIP, surface DIN, and surface currents. Similarly, the width of kelp was significantly positively correlated with surface temperature and salinity and significantly negatively correlated with surface DIP, while the correlations with surface light intensity and surface current were not significant. These results suggested that surface temperature, salinity, DIN, DIP, and currents in Xiangshan Bay had significant impacts on kelp growth. It was worth noting that the lack of significant correlation between surface light intensity and kelp growth parameters was mainly due to the nonlinear effect of light intensity on kelp growth.

### 3.2. Suitability of Physicochemical Factors for Saccharina japonica Growth in Different Cultivation Periods

**Figure 9:**
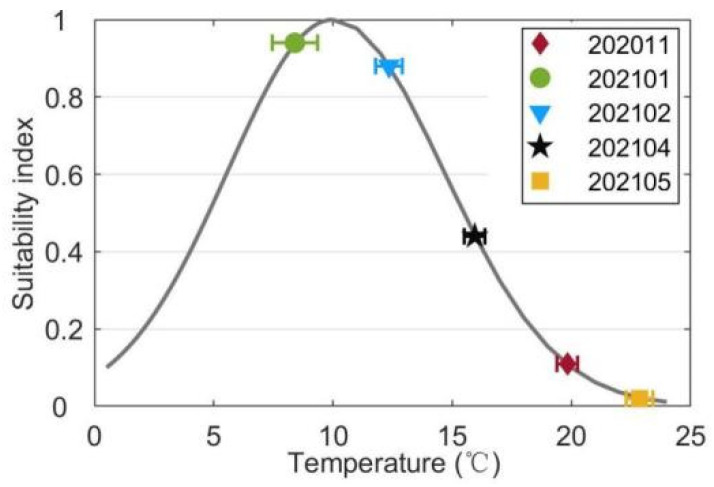
The suitability indices of the average surface temperature collected during each cruise. The horizontal bars represent the standard deviations of the temperature during each cruise.

**Figure 10:**
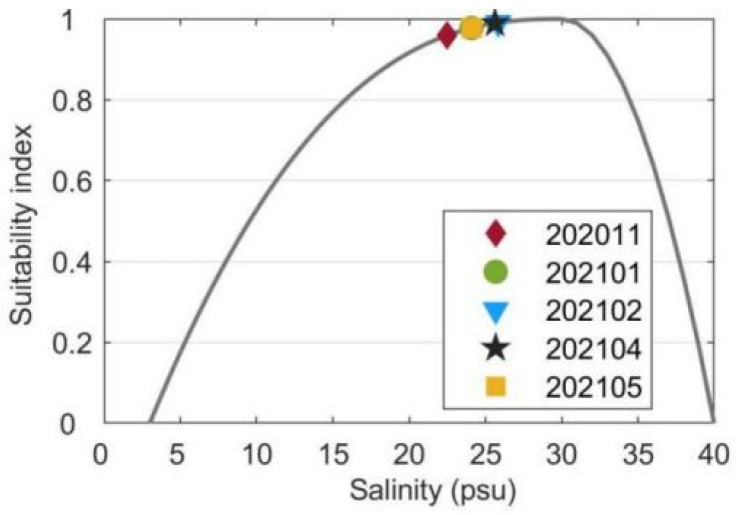
The suitability indices of the average surface salinity collected during each cruise. Note the standard deviations of the surface salinity during each cruise are not plotted in the figure since they are about two orders smaller than the average values.

**Figure 11:**
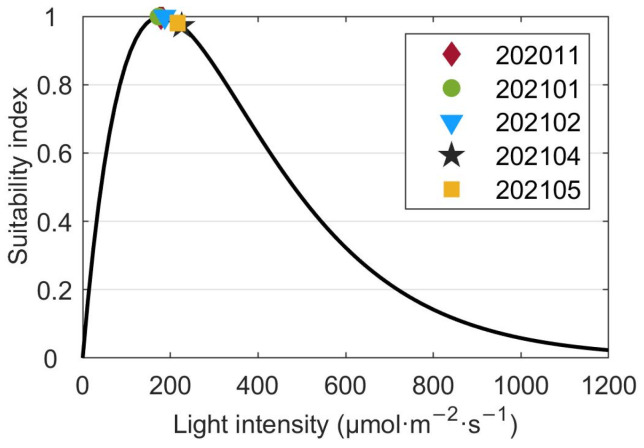
The suitability indices of the average surface light intensity collected during each cruise. Note the standard deviations of the surface light intensity are not calculated as illustrated in the texts.

**Figure 12:**
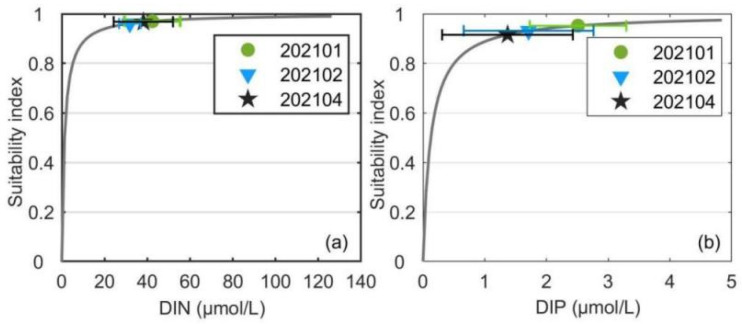
The suitability indices of the DIN (**a**) and DIP (**b**) concentrations during cruises when kelps were cultivated. The horizontal bars represent the standard deviations of the DIN and DIP concentrations during each cruise.

The average surface temperature, salinity, light, DIN and DIP concentrations were substituted into their suitability functions (Equations (1)–(5)). The results showed that the temperature suitability indices during different periods ranged from 0.02 to 0.94 (Figure 9). The suitability indices of the temperature during 202011 and 202105 were as low as 0.11 and 0.02, respectively. Among the three cruises when kelps were cultivated, the suitability index of 202101 was highest with a value of 0.94. Since the standard deviation of temperature was much smaller than the average temperature (Figure 2), the fluctuations of sea surface temperature would not cause considerable drops of the suitability index of temperature during most of the farming period. However, the cold wave during 202101 which caused a drop of the sea surface temperature reduced the suitability index of temperature by about 0.31. The suitability indices of the surface salinity during all cruises varied in the range of 0.96–0.99 (Figure 10). The standard deviations of surface salinity were so small compared to the average salinity that they would not make great drops in the suitability index of salinity (Figure 4). Although the release of terrestrial water from the dam caused a drop of surface salinity by 2.50, the suitability index of salinity only decreased by 0.02. The obtained suitability indices of light intensity varied in the range of 0.97–1.00 (Figure 11). The suitability indices of light intensity during 202011, 202101 and 202102 were all about 1.00. The suitability indices of 202104 and 202105 were decreased to 0.97 and 0.98, respectively. The suitability indices of DIN of each cruise decreased and then increased, ranging from 0.96 to 0.97, while the suitability indices of DIP continued to decrease, ranging from 0.92 to 0.95 (Figure 12).

## 4. Discussion

### 4.1. Implications of the Variation in Prominent Physicochemical Factors for Saccharina japonica Cultivation

The lowest suitability index of the average surface temperature among all cruises was 0.02 (Figure 9), while the suitability indices of other factors during all cruises exceeded 0.92, which showed that temperature was the most critical environmental factor that determined the kelp cultivation time window in Xiangshan Bay. The cultivation time window revealed by the observed temperature in this study was consistent with the kelp cultivation practice in Xiangshan Bay (personal communication with local kelp farmers). The cultivation time window of Sanggou Bay was about eight months [32], when the climate was colder. The time window length in Xiangshan Bay was about three months shorter than that in Sanggou Bay. Therefore, it might be the reason why the kelp harvested in Xiangshan Bay (137.4 × 37.1 cm, Table 1) was much smaller than that in Sanggou Bay (313.0 × 45.0 cm) [41]. Therefore, it is critical to further explore selective breeding of the thermal tolerance variety to increase the kelp yield in South China such as in Xiangshan Bay. Although the size of mature kelp (118.41 ± 16.57 cm) measured by Yang [40] in the same region was similar to our measurement result, our sample size of kelp individuals in each cruise was relatively small. Future studies could increase the sample size to provide more robust results. Some severe weather events during the early and late stages of the farming period when the suitability indices of temperature were inherently low might cause considerable cultivation risks. For example, the cold wave during 202101 caused the suitability index of sea surface temperature to be reduced by about 0.31. In addition, cold waves during the early stage of cultivation and heat waves during the late stage should also be investigated [51,52]. Especially when the water temperature exceeds the maximum physiological threshold of kelp due to marine heat waves, the mortality rate of kelp increases significantly [53]. Therefore, it is necessary to develop high-precision climate models to predict the severe weather events to avoid kelp cultivation risks in South China, and more attention should be paid to the timely harvesting of kelp according to temperature variations during the late stage of kelp cultivation.

The decline of the average DIN concentrations from 202101 to 202102 might be mainly due to the strong absorption of nutrients by the cultivated kelp [36]. The concentrations of DIN and DIP in Xiangshan Bay were affected greatly by terrigenous runoff [44], and the increase in average DIN concentrations from 202102 to 202104 was due to the transition from dry to rainy seasons, during which the terrigenous nutrients loaded into the kelp farm overwhelmed the nutrients absorbed by the cultivated kelp. Unlike DIN, the average DIP concentrations remained decreasing throughout the kelp farming period. In addition to the influence of the high N/P ratio of terrestrial water [50], both phytoplankton and kelp have a high demand for phosphorus in spring, which could cause the DIP concentration to decrease significantly [54]. Therefore, the variation in average DIP concentration in kelp farm might be attributed to the greater consumption by kelp and phytoplankton than supplementation. However, the suitability indices of DIN and DIP during all cruises exceeded 0.92 (Figure 12), even during the late stage of the kelp cultivation, indicating the nutrient conditions were suitable for kelp and would not become a limiting environmental factor at the present scale. To this end, the current kelp cultivation scale did not reach the carrying capacity of Xiangshan Bay, and there is still much potential for development.

The along-bay salinity gradients reversed in direction during 202011, and the reverse of the along-bay salinity gradient was not very unusual in the bay junction as pointed out by Warner et al. [55]. Although there were some variations in the average sea surface salinity between the cruises, the suitability indices of average surface salinity data during all cruises exceeded 0.96. The standard deviations of surface salinity and the dramatic fluctuations during cruise 202105 would not cause great cultivation risks. The average surface light intensity suitability indices were high for all cruises, with the lowest still very close to 1.00. Moreover, the standard deviation of the light intensity was not calculated since the average light intensity was far more important for kelp growth than the fluctuations [56]. In summary, the salinity and light intensity in Xiangshan Bay was very suitable for kelp growth and would not limit the extension of the time window for kelp cultivation. It should be noted that the current research was based on a complete cultivation cycle, which might limit the robustness and generalizability of our findings. Future studies could consider repeating the experiments across more cultivation cycles to further elucidate the effect of environmental conditions on the growth of *Saccharina japonica*.

### 4.2. Assessment of Self-Limiting Effects of Nutrients

The regulation effects of ocean currents on kelp cultivation were usually indirect except when the ocean currents were extremely strong to tear apart kelp blades, such as under typhoon weather [32]. There were few strong winds and waves in Xiangshan Bay [43], so the direct loss of kelp cultivation caused by ocean currents ripping off kelp blades was less. The indirect regulation effects of ocean currents took place through influencing other environmental factors such as nutrients [57] and further caused the self-limiting effects of kelp cultivation. The self-limiting effects due to nutrients could be caused in two ways: (1) the nutrient concentrations became too low to support the proper growth of the abundant kelps during the late stage of the cultivation since the kelps absorbed many nutrients as they grew; (2) the nutrient supplementation from the outer sea to the kelp farm decreased as a result of the weakening circulation caused by the high-density suspended kelp cultivation [39], in turn restricting the further growth of kelp [46].

The ocean currents in coastal seas where kelps were planted usually consisted of periodic tide currents and the quasi-steady circulations. Previous studies observed that the self-limiting effect of kelp cultivation due to nutrients was caused by weakening the circulation and decreasing the related nutrient supplementation in Sanggou Bay [49]. The water exchange was relatively weak due to the obstruction of cultivation facilities and macroalgae in Sanggou Bay kelp farm, and the nutrients were absorbed and could not be replenished in time, so the *Saccharina japonica* yield was significantly affected [58]. Although the surface tide currents in Xiangshan Bay kelp farm were significantly attenuated along with the kelp growth as in Sanggou Bay, the nutrient concentration during the late stage of kelp farming period in Xiangshan Bay was not significantly affected, and the nutrient suitability index remained high. On the one hand, it could be attributed to the fact that the terrigenous water which was rich in nutrients would enhance the nutrient supplements to Xiangshan Bay kelp farm during the rainy season [45]. On the other hand, the offshore residual current had a vital transport effect on environmental factors such as playing a key role in the offshore transportation of nutrients [59], which was crucial for nutrient supplements in the kelp farm. The surface circulation in Xiangshan Bay kelp farm was not weakened by the suspended kelp cultivation. Therefore, the self-limiting effects of the kelp cultivation, which were due to nutrients through weakening circulation and the related nutrient supplements in Xiangshan Bay kelp farm, were not significant.

## 5. Conclusions

The variation characteristics of the five physicochemical environmental factors were analyzed based on the tracking down observations in Xiangshan Bay kelp farm. We found that temperature was the most critical environmental factor that determined the kelp cultivation time window in Xiangshan Bay. The key to increasing the kelp yield in Xiangshan Bay was further selective breeding of the thermal tolerance variety. In addition, the remarkable temperature fluctuations caused by severe weather events during the early and late stages of cultivation would cause great drops in the suitability indices and hence cause cultivation risks. Therefore, developing high-precision climate models to predict the severe weather events is essential to avoid kelp cultivation risks caused by remarkable temperature fluctuations in South China, and more attention should be paid to the timely harvesting of kelp according to temperature variations during the late stage of kelp cultivation. The salinity and light conditions in Xiangshan Bay were very suitable for kelp cultivation. The average salinity and light intensity mainly varied at the season timescale, and their conditions were very suitable for kelp cultivation. Therefore, the salinity and light also did not limit the expansion of the present kelp cultivation time window. The suitability indices of nutrients remained adequate even during the late stage of the kelp cultivation, indicating the self-limiting effects subject to nutrients was not significant in Xiangshan Bay kelp farm. To this end, the present scale of kelp cultivation in Xiangshan Bay did not reach the carrying capacity of the bay, and there is still much potential for development.

The surface tide currents in Xiangshan Bay kelp farm were dramatically attenuated by the suspended kelp cultivation. However, the surface circulation in Xiangshan Bay kelp farm was not suppressed as the kelp growth. The self-limiting effect subject to nutrient supplemented by circulation and the terrigenous water was not significant in Xiangshan Bay.

## Figures and Tables

**Table 1:** Cruise periods and observed data.

Cruise Periods	Referred as in This Paper (yyyymm)	Observed Data					
		Temperature	Salinity	Light	Nutrients	Currents	Kelp Size: Length × Width (cm)
9~16 November 2020	202011	√	√	√	—	√	Before planting
7~15 January 2021	202101	√	√	√	√	√	(24.2 ± 5.9) × (2.8 ± 0.63)
21 February~2 March 2021	202102	√	√	√	√	√	(108.8 ± 8.1) × (27.0 ± 3.7)
6~14 April 2021	202104	√	√	√	√	√	(137.4 ± 15.5) × (37.1 ± 3.2)
27 May~5 June 2021	202105	√	√	√	—	√	After harvest

**Table 2:** Spearman correlation coefficient of kelp size and physicochemical factors.

Parameters	Surface Temperature	Surface Salinity	Surface Light	Surface DIN	Surface DIP	Surface Currents
Kelp length	0.629 *	0.902 **	0.343	−0.629 *	−0.930 **	−0.853 **
Kelp width	0.888 **	0.594 *	0.455	−0.14	−0.671 *	−0.531

Note: **, * represent the significance levels of *p* ≤ 0.01 and *p* ≤ 0.05, respectively.

## Data Availability

The original contributions presented in the study are included in the article, and further inquiries can be directed to the corresponding author.
